# Development and Pilot Testing of a Smartphone-Based Self-Care Program for Patients with Chronic Hepatitis B

**DOI:** 10.3390/ijerph182111139

**Published:** 2021-10-23

**Authors:** Yeonsoo Jang, Sang Hoon Ahn, Kyunghwa Lee, Oh Young Kwon, Jeong Hyun Kim

**Affiliations:** 1 College of Nursing and Mo-Im Kim Nursing Research Institute, Yonsei University, 50-1, Seodaemun-gu, Seoul 03722, Korea; ysjang517@yuhs.ac; 2 College of Medicine, Department of Internal Medicine, Institute of Gastroenterology, Yonsei University, 50-1, Seodaemun-gu, Seoul 03722, Korea; ahnsh@yuhs.ac; 3 College of Nursing, Konyang University, Daejeon 35365, Korea; khlee11@konyang.ac.kr; 4 College of Nursing and Brain Korea 21 FOUR Project, Yonsei University, 50-1, Seodaemun-gu, Seoul 03722, Korea; nursing502@naver.com; 5 College of Nursing, Ansan University, Ansan 15328, Korea

**Keywords:** hepatitis B surface antigens, chronic disease, self-care, mobile applications, quality of life

## Abstract

The purpose of this study is to develop a smartphone-based self-care program (Hep B Care^®^) for patients with the chronic hepatitis B virus (HBV). To pilot test the feasibility of Hep B Care^®^, 63 participants with chronic HBV were recruited from an outpatient clinic at S hospital, Seoul, South Korea (experimental group [EG]: *n* = 30, control group [CG]: *n* = 33) between February and July 2016. Hep B Care^®^ was developed based on the theory of self-care whilst having a chronic illness. During the 12-week intervention period, the application: (1) provided information about the disease, medication, nutrition, and exercise; (2) encouraged taking medication and exercise using alarms; and (3) enabled the exchange of messages between healthcare providers and patients. Salivary cortisol, fatigue, depression, anxiety, knowledge of the HBV, quality of life, and medication adherence were all measured as outcomes. Cortisol levels were significantly increased, knowledge of the HBV was improved, and the mean anxiety score was significantly decreased in the EG. Thus, Hep B Care ^®^ partially improved health outcomes in the EG. We recommend that large trials be conducted among patients with the HBV. The smartphone-based self-care program for providing education and coaching is effective for improving knowledge and reducing anxiety among patients with the HBV.

## 1. Introduction

Hepatitis B is caused by a viral infection. In many cases, patients with the HBV are asymptomatic but, in some cases, they may present symptoms that include jaundice, dark urine, extreme fatigue, nausea, and vomiting. Various symptoms, including acute liver failure or even death, may also occur. The long-term complications of the HBV include liver diseases, such as cirrhosis and hepatocellular carcinoma [1,2]. According to the statistics provided by World Health Organization (WHO), deaths from hepatitis B virus (HBV) in 2015 numbered 887,000 worldwide, most of which were due to cirrhosis and hepatocellular carcinoma [1]. In South Korea, 3.8% of the total population (4.2% males and 3.4% females) is still infected with the HBV [3,4] and approximately 70% of South Korean patients with liver cirrhosis and hepatocellular carcinoma have the HBV surface antigen (HBsAg) [2]. The management of the HBV should be accompanied by lifelong self-care to improve the liver condition of patients and prevent a worsening of the disease [1,2]. However, previous studies have reported that the level of self-care compliance among patients with the HBV is low to moderate [5].

In previous studies, self-care interventions for patients with the HBV were provided using a method of face-to-face group education [6,7]. However, this method is costly and has further disadvantages, such as a deviation of the effects, depending on the level of the provider’s skill and the lack of participant confidentiality [8,9,10]. To address these problems, digital applications (apps) on smart devices provide a new platform for interventions for patients with chronic diseases. Such apps enhance the mutual communication between patients and healthcare providers and are cost-effective [8,10,11]. In addition, the user is able to access the app in real time [12,13]. However, studies on self-care interventions for hepatitis B patients using apps are lacking. The present study differs from prior studies in that we develop an app intervention based on the self-care theory. Therefore, this theory-based study will increase the understanding of the health problems and circumstances of patients with the HBV who need assistance with self-care [14]. In addition, the development process in this study can provide useful information for healthcare providers who create interventions that facilitate self-care for patients with the HBV using the app.

The aims of our study are to: (1) develop a theory-based self-care program using a smartphone app and to (2) test the feasibility of the developed intervention.

## 2. Materials and Methods

This study was conducted in two phases: (1) developing a self-care program (Hep B Care^®,^ Seoul, Korea) using a mobile app for patients with the HBV and (2) pilot testing Hep B Care^®^ for feasibility.

### 2.1. Phase I: Development of a Self-Care Program

#### 2.1.1. Theoretical Basis for Intervention

This app is based on the theory of self-care of patients with a chronic illness [15], which was developed to capture a holistic view of how patients with chronic diseases are treated. Self-care is defined as the process of a patient recognizing and responding to changes in themselves after the acute phase of disease development. There are three key concepts in this theory: self-care maintenance, self-care monitoring, and self-care management. Self-care maintenance comprises the behaviors used by patients with a chronic illness to maintain physical and emotional stability. Self-monitoring is the process through which patients observe themselves to identify the changes in signs and symptoms of the disease. Self-maintenance is defined as the patients’ behaviors for improving personal well-being, preserving their health, or maintaining physical and emotional stability. Riegel et al. [15] also suggest that healthcare professionals provide tailored interventions, including the assurance each patient has easy access to providers/care within the organized healthcare system. These definitions of patients, self-care, and the role of healthcare providers were judged to be the most appropriate for the background and patient context of this study and thus formed the basis of our intervention.

#### 2.1.2. Literature Review for the Selection of Intervention Content

To develop this mobile app, the investigators reviewed the literature in the following way: (a) databases: PubMed, CINAHL Plus with Full Text, EMBASE, KoreaMed, KISS, and RISS; (b) search period: from 2004 to 2016; and (c) keywords: “Hepatitis” [MeSH] or “Hepatitis, Chronic” [MeSH] or “Chronic hepatitis” and “Self Care” [MeSH] or “Self-care” or “Self management” or “Self-management” or “Nonpharmacological intervention” or “Intervention, Nonpharmacological” or “Nursing intervention” or “Intervention, Nursing”.

**Figure 1:**
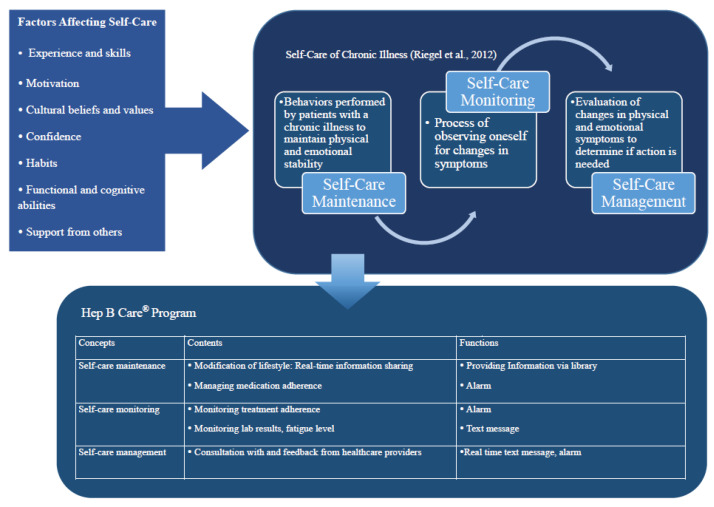
Theoretical framework used in this study.

Through the literature review, we identified the status of the interventions using mobile apps that were available at the time, selected content for the app, and decided on how the app would deliver the content. The intervention content was determined based on the operational definition of the concept in the self-care of chronic illness theory [15]. Detailed information about this theoretical framework is shown in Figure 1.

#### 2.1.3. Expert Content Review

The content of the app was reviewed by an expert group consisting of a physician, two nurses, and two clinical dieticians in a liver clinic. Following the expert review, the development team created a beta version of the mobile app. The beta version was tested at the Department of Gastrointestinal Medicine, in the outpatient clinic at Y University Medical Center, with 10 individuals (5 from the experimental group [EG] and 5 from the control group [CG]), after which the final version was pilot tested.

### 2.2. Phase II: Self-Care Program Pilot Test

#### 2.2.1. Intervention Protocol: EG Versus CG

Participants were randomly assigned to either the EG or the CG using the closed-envelope method. The researcher printed 80 sheets of paper of the same material and size, numbered EG #1 to #40 and CG #1 to #40. The paper was folded to the same size, placed in a white envelope of the same size, and sealed. A research assistant mixed the envelopes and placed them in a box. Patients who agreed to participate in the study were asked to choose one of the sealed envelopes.

The EG installed the self-management of the HBV program app on their mobile phones. The researcher provided training on how to use it. This app provided information regarding the disease process, medication, nutrition, and exercise, and used alarms to encourage users to take medication and exercise. The EG received the knowledge test results on the next day of study-enrollment and learned about the areas in which they lacked knowledge. They also received messages encouraging them to use the app four times during the study period.

The CG was provided with patient education materials and usual healthcare services. Salivary cortisol concentrations were measured after waking up at each time point before the intervention program and 12 weeks later, which was the endpoint of the program. In addition, questionnaires assessing fatigue, quality of life (QOL), depression, and anxiety were administered beforethe beginning of the intervention program and 12 weeks later.

In the planning stage, we did not register the study in the WHO registry network because it was primarily a pilot test of the feasibility of Hep B Care^®^. However, the completed study was registered according to the WHO’s definition of clinical trials (PRE20201213-002).

#### 2.2.2. Setting and Sample

**Figure 2:**
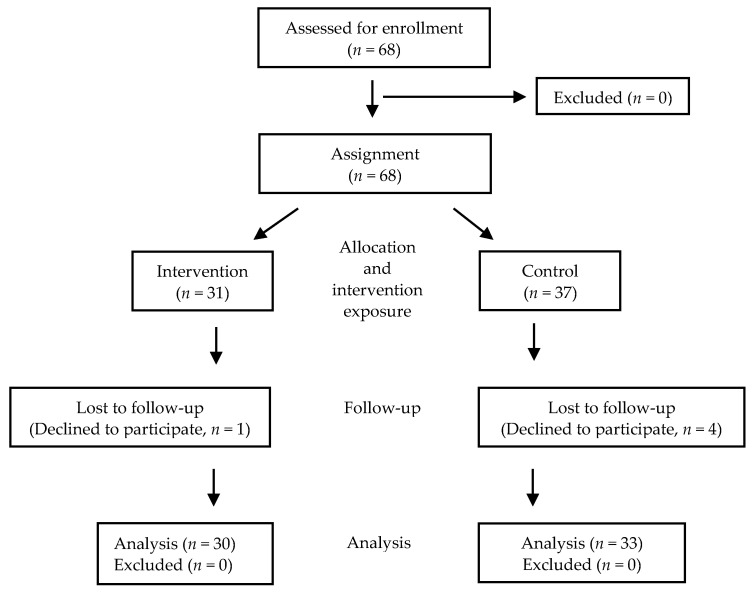
Participant flow.

For this pilot process, the participants were 68 individuals (EG: *n* = 32, CG: *n* = 36) with chronic HBV from an outpatient clinic in Yonsei University Medical Center, in Seoul, South Korea. The eligibility criteria were patients with the HBV: (1) in whom HBsAg had been detected at least six months earlier and the HBV had been diagnosed at least three months earlier; (2) aged > 40 who had not developed liver cirrhosis or cancer; and (3) who had taken an antiviral medication. The exclusion criteria were patients who: (1) were pregnant or had any mental diseases and (2) had other infectious diseases and comorbidities. The flow from enrollment to analysis is illustrated in Figure 2.

#### 2.2.3. Outcome Measurements

The study outcomes were derived as follows through a literature review. Fatigue is the most frequently reported physical symptom in patients with the HBV, and cortisol is a representative biomarker associated with fatigue [16]. Anxiety and depression have also been reported as the most prevalent emotional symptoms in patients with the HBV [17]. Knowledge of disease is the main factor in self-care [18]. Antiviral therapy for chronic HBV is essential for treatment, and patient compliance is a critical factor in antiviral therapy [19]. QOL is widely recognized as an outcome demonstrating the effectiveness of treatment.

Cortisol was obtained from saliva samples drawn in the morning and before bed. The quality control of cortisol ELISA showed intra- and inter-assay coefficients of variations of 6.3% and 10.4%, respectively. The recovery of added cortisol was 101%, and the sensitivity of the assay was 0.071 ng/mL.

To assess knowledge, the investigators evaluated and revised the Level of HBV Knowledge instrument originally developed by Yang (2012) [20]. The revised version consists of 26 items with five subscales: general characteristics of the HBV, infection process, infection prevention, treatment, and management of lifestyle. Participants can answer yes or no, and one point is awarded for each correct answer. The range of the total score is 0–26 points, which is converted to 0–100 points, to help patients to understand their level of knowledge. A high score represents a high knowledge level. The reliability was KR-20 = 0.94 in the original version [20] and KR-20 = 0.75 in this study.

The Korean version of the revised Piper Fatigue Scale was used to measure levels of fatigue [21]. The Piper Fatigue Scale was originally developed in English [22]. The instrument consists of 22 items and four subscales: behavioral/severity, affective meaning, sensory, and cognitive/mood. The total score range is from 0 to 10; a high score represents a high fatigue level. The reliability of the Korean version was α = 0.96 [21]. In this study, the reliability was α = 0.96.

Depression was assessed using the Korean version of the Beck Depression Inventory [23]. It contains 21 items, and the total score ranges from 0 to 63. A high score represents a high level of depression. The reliability of the Korean version was α = 0.93 [23]. In this study, the reliability was α = 0.95.

Anxiety was measured using the Korean version of the Beck Anxiety Scale [24]. It is composed of 21 items, and the score ranges from 0 to 63. A high score represents a high anxiety level. The Korean version showed a reliability of α = 0.93 [25]. In this study, the reliability was α = 0.94.

For QOL, we used the Korean version of the Hepatitis B Quality of Life (HBQOL) questionnaire [26], which consists of 31 items with 6 subscales: psychological well-being, anticipation anxiety, stigma, weakness, vitality, and vulnerability of general health. Each uses a five-point Likert-type scale. The range of total scores is from 0 to 100; a high score represents a high level of QOL. The reliability of the Korean version was α = 0.96 [26]. In this study, the reliability was α = 0.97.

Medication adherence was measured using the Morisky Medication Adherence Scale. This self-report scale was developed to measure the medication adherence in patients with hypertension [27].

#### 2.2.4. Data Collection

We collected data between February and October 2016. The participants’ pre-intervention cortisol levels were measured twice (the evening before and the morning of the intervention); they were also measured twice post-intervention (in the evening and the following morning). Additionally, all participants completed questionnaires before the intervention and again after 12 weeks.

#### 2.2.5. Statistical Analyses

SPSS version 22.0 (IBM Corp., Armonk, NY, USA) was used for the analysis. Descriptive statistics were used to analyze the demographic and disease characteristics of the participants. A chi-square test and an independent *t*-test were used to compare the homogeneity of the two groups. We also performed paired *t*-tests and an ANCOVA to assess the feasibility of this pilot study. The statistical significance level was set at *p* < 0.05.

#### 2.2.6. Ethical Considerations

This study was approved by the Institutional Review Board of a university medical center (IRB#4-2015-1039), and written informed consent was obtained from all participants. Before the study began, the researcher directly explained to the participants the study purpose and procedure; benefits and risks; privacy protection and confidentiality of research data; voluntary nature of participation; and the option to withdraw. Furthermore, the participants were provided with the researcher’s contact information. In the outpatient interview room, which is an independent space, the researcher checked whether the purpose and contents of the study were fully understood along with the explanations above.

## 3. Results

### 3.1. Part I: Development of Hep B Care^®^

#### Content and Functions

In accordance with the literature and guidelines for managing the HBV, the program consisted of a library section for disease information, exercise, diet, and medication; a record section for daily exercise and taking medications; and a Q&A with the provider. We listed self-care practices, including items that had been determined based on empirical knowledge and the literature review concerning patients with the HBV. The self-practice items were arranged according to the self-care of chronic illness theory concepts. For example, self-care maintenance is defined as the “behaviors performed by patients with a chronic illness to maintain physical and emotional stability”. [15] Therefore, the concept of self-care maintenance was narrowed down to a “modification of lifestyle” and “managing medication adherence”. These contents are delivered through the app’s library function, which allows the patient to find the necessary information, and the alarm function, which alerts them to when it is time to take the medication or engage in exercise.

The patient can search for the necessary information, and the library function collects only the information necessary for the individual. In addition, the level of individual knowledge about chronic HBV can be measured in the app, and the administrator can be notified of the topic for which the patient needs further information. Also, self-care monitoring is defined as the “process of observing oneself for changes in symptoms” [15]. We narrowed that definition down to “monitoring treatment adherence”, “monitoring lab results”, and “monitoring fatigue level” for the purposes of the app. Alarms are set to notify patients of their upcoming scheduled outpatient visits (alerts are provided one week before and one day before the scheduled appointment dates) and if there are lab results that show abnormal values, the patient is sent a notification via text.

In the self-care chronic illness theory, self-care management is defined as the “evaluation of changes in physical and emotional symptoms to determine if action is needed” [15]. For use in the smartphone app, this concept was narrowed down to a “consultation with/feedback from healthcare providers.” Participants could ask questions related to self-care via text message and look up their history of self-management in any given week or month.

**Figure 3:**
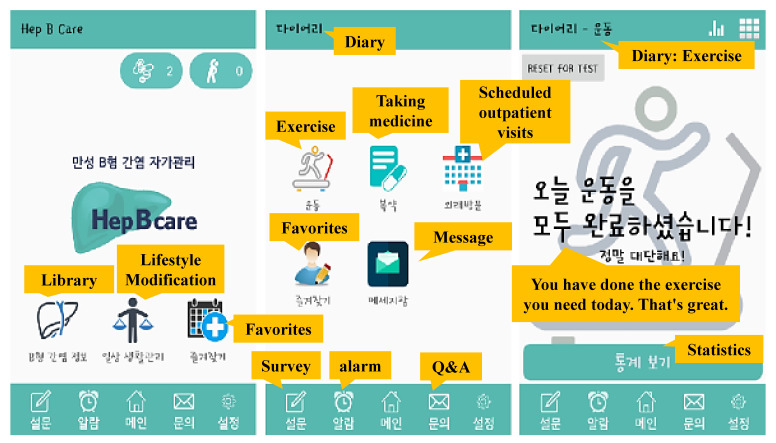
Images of Hep B Care^®^.

The final version of the app was developed after modifying the beta version based on participants’ experiences and feedback. In the final version, technical errors were rectified, and the following content was added: brand and generic names of medications and foods for high-protein diets (Figure 3). We named the program “Hep B Care^®^”.

### 3.2. Part II: Pilot Study of Feasibility of the Hep B Care^®^ Program

Pilot study analyses revealed that there were no statistical differences in the baseline characteristics between the EG and CG. The mean (standard deviation) age was 55.0 (7.35) years in the EG and 52.3 (7.35) years in the CG. In each group, 21 patients (70% and 63.6%, respectively) were male (Table 1).

#### 3.2.1. Results by Measured Outcomes

##### Cortisol

EG participants’ cortisol levels were significantly increased at week 12 (*p* = 0.037) compared to pre-intervention (Table 2). However, when pre-cortisol values were adjusted, there was no significant difference between the groups (Table 3).

##### Knowledge

EG participants’ knowledge levels were significantly increased at week 12 (*p* = 0.009) compared to pre-intervention (Table 2). In the subdomains, the “knowledge of transmission” had significantly increased in the EG (*p* = 0.011) (Table 2). When the pre-knowledge values were adjusted, we found a significant difference between the EG post-intervention scores and the CG scores (*p* = 0.004). Also the “knowledge of general characteristics”, “knowledge of transmission”, “knowledge of infection process”, and “knowledge of treatment” had significantly increased in the EG (Table 3).

##### Fatigue

There was no significant difference in pre- and post-fatigue between the two groups, and fatigue did not affect the intervention’s effectiveness (Table 2 and Table 3).

##### Depression

There was no significant difference in pre- and post-depression between the two groups, and depression did not affect the intervention’s effectiveness (Table 2 and Table 3).

##### Anxiety

EG participants’ anxiety levels were significantly increased at week 12 (*p* = 0.038) compared to pre-intervention (Table 2). When the pre-anxiety values were adjusted, we found a significant difference between the EG post-intervention scores and the CG scores (*p* = 0.006) (Table 3).

##### Quality of Life (QOL)

There was no significant difference in pre- and post-QOL between the two groups, and QOL did not affect the intervention’s effectiveness (Table 2 and Table 3).

##### Medication Adherence

There was no significant difference in pre- and post-medication adherence between the two groups, and the medication adherence did not affect the intervention’s effectiveness (Table 2 and Table 3).

## 4. Discussion

Patients with chronic HBV need to perform self-care throughout their lifetime. After the treatment of acute symptoms, healthcare providers need to ensure that patients are protected from worsening complications of the liver disease by encouraging them to engage in infection prevention by taking regular medication, exercising, and performing dietary management. In this study, we developed Hep B Care^®^ as a self-care program using a mobile app and pilot tested its feasibility. The Hep B Care^®^ program has the following three characteristics.

First, the component of knowledge about the HBV is highlighted. In previous studies, healthcare providers educated participants about the HBV using traditional approaches, such as seminars [28,29]. Conversely, Hep B Care^®^ provides knowledge in a more interactive way. Specifically, participants can receive immediate feedback after assessing their level of knowledge and review the material anytime; thus, this system can provide tailored information and may improve participation in an intervention program. In studies targeting other diseases, such as cancer, or for the health promotion of the general population, participants’ knowledge has been shown to increase more when an intervention includes feedback, such as showing them a message that confirms their status [30,31].

Second, our program emphasizes interactive communication. Participants in the pilot study could send messages with their questions to their healthcare providers whenever they wanted. Previous studies have shown that the interaction between patients with diabetes mellitus and the healthcare providers positively affected their self-care [32,33]. Given that patient engagement is essential to improving patient outcomes [34], the interaction enabled by Hep B Care^®^ might increase the patients’ engagement in the intervention.

Third, the investigators sent encouraging messages to patients four times via this program. Compliance is another factor known to influence the effectiveness of treatment [35], and encouragement from the healthcare provider is known to have a major impact on adherence [36]. Hence, previous studies have also attempted to increase the participants’ compliance through encouraging messages [37,38].

Through the Hep B Care^®^ program with the above characteristics, the following three key findings were obtained in this pilot study. First, cortisol levels in the EG were significantly increased after the intervention, compared to the cortisol levels before the intervention. Although the Hep B Care^®^ program had no significant effect on perceived fatigue in the EG, their cortisol levels changed significantly. A previous study on Korean patients with the HBV reported that patients with higher fatigue levels had lower levels of cortisol [16]. This pattern of cortisol is similar to that of chronic fatigue syndrome [39]. To better assess the effects of the Hep B Care^®^ program on fatigue as one of the major symptoms in individuals with the HBV, further studies will need to be conducted with objective outcome indicators using a wider range of biomarkers.

Second, the EG demonstrated a significant improvement in knowledge and a reduction in anxiety, despite the small change in the mean scores. The participants’ level of knowledge in this study was relatively high, which is thought to be due to the long time since being diagnosed with HBVand the high level of education. Enhancing the patients’ knowledge of these chronic diseases is necessary to ensure the intervention addresses any shortcomings (rather than simply improving their overall scores). Meanwhile, patients with chronic HBV have anxiety about infection and the deterioration of liver function, and these forms of mental stress appear to negatively impact their QOL [40,41]. Although there have been few intervention studies in patients with the HBV, randomized controlled trial studies with patients with chronic illness have shown that educational interventions reduce anxiety [42,43]. Since anxiety in these studies was assessed with a general instrument, there might have been a limitation to explaining the disease-specific anxiety experienced by patients with the HBV. Therefore, it is necessary to identify the characteristics of anxiety in patients with the HBV in further research.

Third, HBQOL was not significantly different after the intervention in this study. It is known that self-care intervention improves the health-related quality of life (HRQOL) in patients with chronic diseases [44,45]. Self-care for managing physical symptoms can lead to an improvement in HRQOL in patients with chronic illnesses [46]. However, the level of fatigue as a major symptom was not significantly different in this study. This could be the reason why the HBQOL was not significantly different after the intervention. Therefore, the relationship between fatigue management and the HBQOL needs to be clarified in this population.

Our findings show that this tailored intervention using a smartphone app for patients with the HBV is feasible. There are few mobile interventions targeting this population, so the current program may help to improve the self-care behaviors of the patients. Furthermore, the participants could access the mobile system and communicate with providers without the limitations of time and place. Since most patients are also employed, they may find it burdensome to receive education through hospital visits, owing to time and place constraints. However, this study has several limitations that should be noted. First, we did not evaluate the satisfaction of the patients with the app. User satisfaction needs to be surveyed in the main study. Second, a bias may have occurred in the process of collecting the cortisol. We did not closely monitor the participants; instead, we gave them instructions for collecting cortisol by themselves. In addition, although the group assignment was randomized, a performance bias could exist because the EG could guess that they were receiving the intervention, and the intervention provider could also know to which group the patients belonged. Therefore, the results should be interpreted with caution. Lastly, this study is a pilot study conducted with a small sample size. While chronic hepatitis B is usually a disease that requires lifelong treatment and follow-up, our study was conducted over a short period of time and the long-term effectiveness of the intervention cannot be determined. Therefore, larger samples and longer-term follow-up studies should be conducted to further evaluate the effectiveness of this program.

## 5. Conclusions

The findings of this pilot test indicate that the Hep B Care^®^ program is a feasible intervention for self-care for South Korean patients with chronic HBV. This mobile health self-care program may be effective for improving self-care in this population because it can provide tailored intervention based on the patients’ care needs. In addition, mobile health intervention is known to be effective for improving self-care in middle-aged populations familiar with receiving and exchanging information, including health information, via smartphones. Finally, the results of this pilot study indicate that the Hep B Care^®^ program is feasible for improving the patients’ knowledge of the HBV and reducing their anxiety levels.

## Figures and Tables

**Table 1:** Demographic characteristics of participants in a pilot test of a smart app for HBV management. N = 63.

Characteristics		Experimental (n = 30)		Control (n = 33)			
		Mean ± SD or n (%)				*χ*^2^ or *t*	*p*
Age, years		55.0 ± 7.35		52.3 ± 7.35		1.446	0.153
Sex	Male	21	(70.0)	21	(63.6)	0.286	0.789
	Female	9	(30.0)	12	(36.4)		
Marital status	Married	27	(90.0)	32	(97.0)	1.284	0.340
	Single or widowed	3	(10.0)	1	(3.0)		
Occupation	Employed	15	(50.0)	22	(66.7)	1.801	0.208
	Unemployed	15	(50.0)	11	(33.3)		
Education	≤High school	11	(36.7)	17	(51.5)	1.403	0.312
	>High school	19	(63.3)	16	(48.5)		
Household income (Month, KRW)	<2 million	4	(13.4)	3	(9.1)	3.507	0.676
	2–3 million	3	(10.0)	6	(18.2)		
	3–4 million	5	(16.7)	3	(9.1)		
	4–5 million	7	(23.2)	8	(24.2)		
	5–6 million	11	(36.7)	13	(39.4)		
Length of diagnosis (months)		90.8 ± 55.06		97.1 ± 84.69		−0.242	0.811
Hospitalization	Yes	6	(20.0)	7	(21.2)	0.014	1.000
	No	24	(80.0)	26	(78.8)		
Family history of HBV	Yes	23	(76.7)	25	(75.8)	0.007	1.00
	No	7	(23.3)	8	(24.2)		
Comorbidity	Yes	7	(23.3)	10	(30.3)	0.387	0.581
	No	23	(76.7)	23	(69.7)		
Taking antivirals	Yes	30	(100.0)	33	(100.0)		
Duration of taking antivirals (months)		88.6 ± 60.47		87.6 ± 48.90		0.069	0.945
HBV DNA	Target not detected	11	(36.7)	9	(27.3)	2.350	0.329
	<20	14	(46.7)	13	(39.4)		
	≥20	5	(16.6)	11	(33.3)		
AST		27.3 ± 15.98		24.4 ± 9.07		0.908	0.367
ALT		24.2 ± 13.42		26.6 ± 21.9		−0.526	0.601

Comorbidity: hypertension, diabetes mellitus, hyperlipidemia, and atherosclerosis, etc.; App: application; ALT: alanine aminotransferase; AST: aspartate aminotransferase; DNA: deoxyribonucleic acid; HBV: hepatitis B virus; KRW: Korean Won; and SD: standard deviation.

**Table 2:** Comparison of outcomes of groups in a 12-week pilot test of a smart app intervention for HBV management. N = 63.

Variables		Experimental Group (n = 30)						Control Group (n = 33)					
		Pre		Post				Pre		Post			
		Mean ± SD				*t*	(*p*)	Mean ± SD				*t*	(*p*)
Cortisol		1.90	±0.44	2.02	±0.44	−2.19	(0.037 *)	1.47	±0.62	1.35	±0.48	1.03	(0.313)
Knowledge	Total	78.98	±11.39	83.65	±1.49	−2.81	(0.009 *)	74.92	±13.66	77.39	±16.11	−1.47	(0.152)
	GE	89.29	±24.09	94.05	±13.00	−1.00	(0.326)	87.69	±18.19	90.91	±20.87	−1.21	(0.235)
	TM	77.23	±18.34	85.71	±13.91	−2.75	(0.011 *)	74.62	±19.13	77.27	±21.30	−0.96	(0.344)
	IP	72.62	±19.88	75.00	±15.38	−0.61	(0.547)	62.12	±20.53	65.15	±23.33	−0.97	(0.338)
	TR	89.29	±12.74	89.29	±13.86	0	(1.000)	85.45	±13.48	86.06	±15.40	−0.20	(0.845)
	DA	71.43	±16.27	77.68	±20.79	−1.57	(0.129)	71.97	±19.52	75.00	±22.53	−0.75	(0.458)
Fatigue		4.07	±2.50	4.09	±2.70	−0.11	(0.914)	3.83	±2.21	3.68	±2.00	0.78	(0.442)
Depression		10.23	±10.75	9.73	±10.66	0.44	(0.663)	8.36	±8.82	7.18	±6.78	1.08	(0.286)
Anxiety		30.27	±10.04	27.90	±9.01	2.17	(0.038 *)	29.24	±8.70	27.15	±4.96	1.67	(0.101)
HBQOL	Total	63.31	±22.60	65.43	±22.05	−1.02	(0.317)	67.28	±21.41	66.79	±20.23	0.25	(0.807)
	PW	76.94	±25.38	77.69	±25.05	−0.25	(0.806)	78.79	±24.29	79.71	±23.69	−0.45	(0.658)
	AA	43.06	±24.01	48.06	±28.34	−1.85	(0.075)	48.23	±25.79	44.57	±26.32	0.98	(0.333)
	ST	65.00	±28.42	65.42	±26.74	−0.16	(0.874)	69.82	±22.54	70.58	±23.01	−0.29	(0.771)
	WN	78.89	±25.31	78.89	±24.64	0	(1.000)	80.30	±25.41	84.60	±20.21	−1.18	(0.248)
	VI	64.58	±26.48	70.00	±29.43	−1.58	(0.125)	68.18	±24.67	66.86	±22.34	0.46	(0.652)
	VU	42.22	±24.07	43.89	±26.98	−0.46	(0.651)	51.52	±29.86	46.97	±27.31	0.92	(0.363)
Medication adherence		1.20	± 1.10	1.17	±1.05	0.25	(0.801)	1.52	±1.12	1.24	±1.03	1.39	(0.174)

* *p* < 0.05, App: application; HBV: hepatitis B virus; GE: general characteristics; TM: transmission; IP: infection process; TR: treatment; DA: daily management of lifestyle; PW: psychological well-being; AA: anticipation anxiety; ST: stigmatization; WN: weakness; VI: vitality; and VU: vulnerability.

**Table 3:** Mean differences in outcomes after a 12-week pilot test of a smart app intervention for HBV management. N = 63.

Variables		Range	Experimental (n = 30)	Control (n = 33)	F (*p*)	
			Pre-Post	Pre-Post		
			LS Mean ± SE			
Cortisol			1.85 ± 0.08	1.39 ± 0.07	1.68	(0.200)
Knowledge	Total	0–100	82.68 ± 1.61	79.15 ± 1.47	9.15	(0.004 *)
	GE	0–100	93.96 ± 2.68	91.50 ± 2.47	16.09	(<0.001 *)
	TM	0–100	85.16 ± 2.67	78.20 ± 2.46	5.51	(0.022 *)
	IP	0–100	73.52 ± 3.21	68.84 ± 2.92	6.59	(0.013 *)
	TR	0–100	87.97 ± 2.60	86.58 ± 2.38	1.39	(0.243)
	DA	0–100	77.82 ± 3.86	74.89 ± 3.55	0.008	(0.931)
Fatigue		0–10	3.98 ± 0.22	3.77 ± 0.21	0.43	(0.514)
Depression		0–63	8.93 ± 0.99	7.67 ± 0.95	0.49	(0.487)
Anxiety		0–63	27.51 ± 0.87	27.31 ± 0.83	8.18	(0.006 *)
HBQOL	Total	0–100	67.20 ± 2.00	65.27 ± 1.91	0.01	(0.919)
	PW	0–100	78.44 ± 2.50	78.96 ± 2.38	0.22	(0.640)
	AA	0–100	50.78 ± 3.28	42.90 ± 3.12	0.57	(0.452)
	ST	0–100	67.48 ± 2.56	68.73 ± 2.45	0.43	(0.836)
	WN	0–100	79.42 ± 3.06	84.28 ± 2.92	2.80	(0.100)
	VI	0–100	71.63 ± 3.09	65.69 ± 2.95	0.29	(0.594)
	VU	0–100	47.70 ± 4.09	44.89 ± 3.86	1.13	(0.292)
Medication adherence		1–4	1.29 ± 0.15	1.18 ± 0.14	1.04	(0.312)

* *p* < 0.05, App: application; HBV: hepatitis B virus; SE: standard error; GE: general characteristics; TM: transmission; IP: infection process; TR: treatment; DA: daily management of lifestyle; PW: psychological well-being; AA: anticipation anxiety; ST: stigmatization; WN: weakness; VI: vitality; and VU: vulnerability.

## Data Availability

All relevant data are available from the Figshare database at doi:10.6084/m9.figshare.16601345.

## References

[1] World Health Organization Hepatitis b. https://www.who.int/news-room/fact-sheets/detail/hepatitis-b.

[2] Korean Association for the Study of the Liver (2019). Kasl clinical practice guidelines for management of chronic hepatitis b. Clin. Mol. Hepatol..

[3] Korean Society for The Study of Obesity (2018). 2018 Clinical Practice Guidelines for Overweight and Obesity in Korea.

[4] Korea Statistical Information Service Trend in Seroprevalence of Hepatitis b Surface Antigen(hbs ag) in Korea: Sex, 10 Years or Older. http://kosis.kr/statHtml/statHtml.do?orgId=117&tblId=DT_11702_N109.

[5] Seo I.S., Song M.J., Yoo Y.S., Kim H.S. (2017). Comparison for hepatitis b knowledge, self care practice and quality of life according to the disease activity among patients with the hepatitis b virus. J. Korean Public Health Nur..

[6] Cui Y., Moriyama M., Chayama K., Liu Y., Ya C., Muzembo B.A., Rahman M.M. (2019). Efficacy of a self-management program in patients with chronic viral hepatitis in china. BMC Nurs..

[7] Hyun C.S., Ventura W.R., Kim S.S., Yoon S., Lee S. (2016). A community- based hepatitis b linkage-to-care program: A case study on asian americans chronically infected with hepatitis b virus. Hepatol. Med. Policy.

[8] Iribarren S.J., Cato K., Falzon L., Stone P.W. (2017). What is the economic evidence for mhealth? A systematic review of economic evaluations of mhealth solutions. PLoS ONE.

[9] McPherson S., Wicks C., Tercelli I. (2020). Patient experiences of psychological therapy for depression: A qualitative metasynthesis. BMC Psychiatry.

[10] Jiang X., Ming W.K., You J.H. (2019). The cost-effectiveness of digital health interventions on the management of cardiovascular diseases: Systematic review. J. Med. Internet Res..

[11] Guerriero C., Cairns J., Roberts I., Rodgers A., Whittaker R., Free C. (2013). The cost-effectiveness of smoking cessation support delivered by mobile phone text messaging: Txt2stop. Eur. J. Health Econ..

[12] Dennison L., Morrison L., Conway G., Yardley L. (2013). Opportunities and challenges for smartphone applications in supporting health behavior change: Qualitative study. J. Med. Internet Res..

[13] Turner-McGrievy G.M., Beets M.W., Moore J.B., Kaczynski A.T., Barr-Anderson D.J., Tate D.F. (2013). Comparison of traditional versus mobile app self-monitoring of physical activity and dietary intake among overweight adults participating in an mhealth weight loss program. J. Am. Med. Inform. Assoc..

[14] Heath G., Cooke R., Cameron E. (2015). A theory-based approach for developing interventions to change patient behaviours: A medication adherence example from paediatric secondary care. Healthcare.

[15] Riegel B., Jaarsma T., Stromberg A. (2012). A middle-range theory of self-care of chronic illness. ANS Adv. Nurs. Sci..

[16] Jang Y., Kim J.H., Lee H., Lee K., Ahn S.H. (2018). A quantile regression approach to explain the relationship of fatigue and cortisol, cytokine among koreans with hepatitis b. Sci. Rep..

[17] Li G., Wang G., Hsu F.C., Xu J., Pei X., Zhao B., Shetty A. (2020). Effects of depression, anxiety, stigma, and disclosure on health-related quality of life among chronic hepatitis b patients in dalian, China. Am. J. Trop. Med. Hyg..

[18] Orem D.E. (1985). A concept of self-care for the rehabilitation client. Rehabil. Nurs..

[19] European Association for the Study of the Liver (2017). Easl 2017 clinical practice guidelines on the management of hepatitis b virus infection. J. Hepatol..

[20] Yang J.H. (2012). Development and evaluation of a program to promote self management in patients with chronic hepatitis b. J. Korean Acad. Nurs..

[21] Jang Y., Kim J.H., Lee K. (2017). Validation of the revised piper fatigue scale in koreans with chronic hepatitis b. PLoS ONE.

[22] Piper B.F., Dibble S.L., Dodd M.J., Weiss M.C., Slaughter R.E., Paul S.M. (1998). The revised piper fatigue scale: Psychometric evaluation in women with breast cancer. Oncol. Nurs. Forum.

[23] Lee M.K., Lee Y.H., Park S.H., Sohn C.H., Jung Y.J., Hong S.K., Lee B.G., Jang P., Yun A. (1995). A standardization study of beck depression inventory (i): Korean version (k-bdi): Reliability land factor analysis. J. Psychopathol..

[24] Kwon S.-M. (1992). Differential Roles of Dysfunctional Attitudes and Automatic Thoughts in Depression: An Integrated Cognitive Model of Depression.

[25] Kwon S.M., Oei T.P. (1992). Differential causal roles of dysfunctional attitudes and automatic thoughts in depression. Cognit. Ther. Res..

[26] Jang Y., Ahn S.H., Lee K., Lee J., Kim J.H. (2019). Psychometric evaluation of the korean version of the hepatitis b quality of life questionnaire. PLoS ONE.

[27] Morisky D.E., Green L.W., Levine D.M. (1986). Concurrent and predictive validity of a self-reported measure of medication adherence. Med. Care.

[28] Robotin M., Patton Y., George J. (2013). Getting it right: The impact of a continuing medical education program on hepatitis b knowledge of australian primary care providers. Int. J. Gen. Med..

[29] Koman D. (2018). Increasing hepatitis c virus knowledge through an evidence-based educational intervention. Gastroenterol. Nurs..

[30] Wojcikowski K., Kirk L. (2013). Immediate detailed feedback to test-enhanced learning: An effective online educational tool. Med. Teach..

[31] Schwartz L.A., Daniel L.C., Henry-Moss D., Bonafide C.P., Li Y., Psihogios A.M., Butler E.S., Szalda D., Ver Hoeve E.S., Hobbie W.L. (2020). Feasibility and acceptability of a pilot tailored text messaging intervention for adolescents and young adults completing cancer treatment. Psychooncology.

[32] Wu X., Guo X., Zhang Z. (2019). The efficacy of mobile phone apps for lifestyle modification in diabetes: Systematic review and meta-analysis. JMIR Mhealth Uhealth.

[33] Gong E., Baptista S., Russell A., Scuffham P., Riddell M., Speight J., Bird D., Williams E., Lotfaliany M., Oldenburg B. (2020). My diabetes coach, a mobile app-based interactive conversational agent to support type 2 diabetes self-management: Randomized effectiveness-implementation trial. J. Med. Internet Res..

[34] World Health Organization Patient Engagement. https://apps.who.int/iris/bitstream/handle/10665/252269/9789241511629-eng.pdf;jsessionid=F52633A53EF3C99D0FF6107A9641B70C?sequence=1.

[35] Jin J., Sklar G.E., Oh V.M.S., Li S.C. (2008). Factors affecting therapeutic compliance: A review from the patient’s perspective. Ther. Clin. Risk Manag..

[36] Lin W.S., Lee T.T., Yang Y.H., Mills M.E. (2019). Environmental factors affecting self-management of chronic hepatitis b from the patients’ perspective. J. Clin. Nurs..

[37] Thakkar J., Kurup R., Laba T.L., Santo K., Thiagalingam A., Rodgers A., Woodward M., Redfern J., Chow C.K. (2016). Mobile telephone text messaging for medication adherence in chronic disease: A meta-analysis. JAMA Intern. Med..

[38] Mayer J.E., Fontelo P. (2017). Meta-analysis on the effect of text message reminders for hiv-related compliance. AIDS Care.

[39] De Bellis A., Bellastella G., Pernice V., Cirillo P., Longo M., Maio A., Scappaticcio L., Maiorino M.I., Bellastella A., Esposito K. (2021). Hypothalamic-pituitary autoimmunity and related impairment of hormone secretions in chronic fatigue syndrome. J. Clin. Endocrinol. Metab..

[40] Fotos N.V., Elefsiniotis I., Patelarou A., Giakoumidakis K., Patelarou E., Kouros A., Brokalaki H. (2018). Psychological disorders and quality of life among patients with chronic viral hepatitis: A single-center cross-sectional study with pair-matched healthy controls. Gastroenterol. Nurs..

[41] Keskin G., Gumus A.B., Orgun F. (2013). Quality of life, depression, and anxiety among hepatitis b patients. Gastroenterol. Nurs..

[42] Lemos M.F., Lemos-Neto S.V., Barrucand L., Verçosa N., Tibirica E. (2019). Preoperative education reduces preoperative anxiety in cancer patients undergoing surgery: Usefulness of the self-reported beck anxiety inventory. Braz. J. Anesthesiol..

[43] Petricone-Westwood D., Jones G., Mutsaers B., Leclair C.S., Tomei C., Trudel G., Dinkel A., Lebel S. (2019). A systematic review of interventions for health anxiety presentations across diverse chronic illnesses. Int. J. Behav. Med..

[44] Abbasi A., Ghezeljeh T.N., Farahani M.A. (2018). Effect of the self-management education program on the quality of life in people with chronic heart failure: A randomized controlled trial. Electron. Physician.

[45] Xu X., Han J., Li Y., Sun X., Lin P., Chen Y., Gao F., Li Z., Zhang S., Sun W. (2020). Effects of orem’s self-care model on the life quality of elderly patients with hip fractures. Pain Res. Manag..

[46] Ahn S., Song R., Choi S.W. (2016). Effects of self-care health behaviors on quality of life mediated by cardiovascular risk factors among individuals with coronary artery disease: A structural equation modeling approach. Asian Nurs. Res..

